# Immunological Similarities and Differences between Post-COVID-19 Lung Sequelae and Idiopathic Pulmonary Fibrosis

**DOI:** 10.3390/biomedicines12030630

**Published:** 2024-03-12

**Authors:** Sara Gangi, Laura Bergantini, Paolo Cameli, Irene Paggi, Marco Spalletti, Fabrizio Mezzasalma, Elena Bargagli, Miriana d’Alessandro

**Affiliations:** 1 Respiratory Diseases and Lung Transplantation Unit, Department of Medical and Surgical Sciences & Neuro-Sciences, University of Siena, 53100 Siena, Italy; sara.gangi@student.unisi.it (S.G.); laurabergantini@gmail.com (L.B.); paolocameli88@gmail.com (P.C.); irene.paggi@student.unisi.it (I.P.); marcospalletti@live.it (M.S.); bargagli2@gmail.com (E.B.); 2 Diagnostic and Interventional Bronchoscopy Unit, Cardio-Thoracic and Vascular Department, University Hospital of Siena, Azienda Ospedaliera Universitaria Senese—AOUS, 53100 Siena, Italy; fabriziomezzasalma@hotmail.it

**Keywords:** idiopathic pulmonary fibrosis, post-COVID pulmonary fibrosis, immune cells, immune checkpoint, CD4, CD8, NK, PD-1, TIGIT

## Abstract

Introduction: Pulmonary fibrosis is an irreversible condition that may be caused by known (including viral triggers such as SARS-CoV-2) and unknown insults. The latter group includes idiopathic pulmonary fibrosis (IPF), which is a chronic, progressive fibrosing interstitial pneumonia of unknown cause. The longer the insult acts on lung tissue, the lower the probability of a complete resolution of the damage. An emerging clinical entity post-COVID-19 is pulmonary fibrosis (PCPF), which shares many pathological, clinical, and immunological features with IPF. The fibrotic response in both diseases—IPF and PCPF—is orchestrated in part by the immune system. An important role regarding the inhibitory or stimulatory effects on immune responses is exerted by the immune checkpoints (ICs). The aim of the present study was to analyse the similarities and differences between CD4+, CD8+, and NK cells in the peripheral blood of patients affected by fibrotic disease, IPF, and PCPF compared with sarcoidosis patients and healthy controls. The second aim was to evaluate the expression and co-expression of PD-1 and TIGIT on CD4, CD8, and NK cells from our patient cohort. Methods: One hundred and fifteen patients affected by IPF, PCPF, and sarcoidosis at the rare pulmonary disease centre of the University of Siena were enrolled. Forty-eight patients had an IPF diagnosis, 55 had PCPF, and 12 had sarcoidosis. Further, ten healthy controls were enrolled. PCPF patients were included between 6 and 9 months following hospital discharge for COVID-19. The peripheral blood samples were collected, and through flow cytometric analysis, we analysed the expression of CD4, CD8, NK cells, PD-1, and TIGIT. Results: The results show a greater depletion of CD4 and NK cells in IPF patients compared to other groups (*p* = 0.003), in contrast with CD8 cells (*p* < 001). Correlation analysis demonstrated an indirect correlation between CD4 and CD8 cells in IPF and sarcoidosis patients (*p* < 0.001 = −0.87 and *p* = 0.042; r = −0.6, respectively). Conversely, PCPF patients revealed a direct correlation between CD4 and CD8 cells (*p* < 0.001; r = 0.90) accentuating an immune response restoration. The expression of PD-1 and TIGIT was abundant on T and NK cell subsets of the two lung fibrotic groups, IPF and PCPF. Analogously, the co-expression of PD-1 and TIGIT on the surfaces of CD4 and CD8 were increased in such diseases. **Conclusions**: Our study shines a spotlight on the immune responses involved in the development of pulmonary fibrosis, idiopathic and secondary to SARS-CoV-2 infection. We observed a significant imbalance not only in CD4, CD8, and NK blood percentages in IPF and PCPF patients but also in their functional phenotypes evaluated through the expression of ICs.

## 1. Introduction

Pulmonary fibrosis is an irreversible condition characterised by scarring and thickening of the lung interstitium that leads to impaired gas transfer, loss of lung function, and in many cases, death. It may be caused by known (including viral triggers such as SARS-CoV-2) and unknown insults. The latter include idiopathic pulmonary fibrosis (IPF) and sarcoidosis. IPF is a chronic progressive idiopathic interstitial lung disease (ILD) [1] of unclear pathogenesis. Recurrent ongoing injury to alveolar epithelial cells triggers release of proinflammatory mediators (such as Transforming growth factor beta, TGF-β) and accumulation of immune and profibrotic cells in the lung, accompanied by deposition of a large amount of extracellular matrix (ECM). Moreover, pulmonary cellular damage induced by several factors (environmental, infections, mechanical damage) results in the disruption of lung parenchymal architecture. In this compromise microenvironment, resident and recruited immune cells such as macrophages and lymphocytes modulate existing responses through a variety of mechanisms [2]. These abnormalities contribute to the development and progression of IPF (ref [3]). Altered proportion or activation of T cells subsets, as well as specific receptors, were shown to negatively influence the progression of this disease [4]. 

T cells are diffusely present in the alveoli, lung tissues, and bloodstream of IPF patients, though their role is still controversial. CD4 and CD8 cells are both involved in the progression of the IPF. CD4 cell subsets play a profibrotic role in its pathogenesis, producing IL-4, IL-5 and IL-13 [5]. CD8 cells may impact the development of pulmonary fibrosis, infiltrating the lung parenchyma through the release of cytokines. Controversially, Koh et al. reported that CD8 cells may produce cytokines with pro- and anti-fibrotic properties [6]. Croft et al. demonstrated that an increased CD8 cell percentages was associated with severe lung injury [7]. An altered immune system has been reported in hospitalised COVID-19 patients, though few data are available about its impairment in the follow-up. 

Post-COVID-19 pulmonary fibrosis (PCPF) is an emerging clinical entity following SARS-CoV-2 infection that shares many pathological, clinical, and immunological features with IPF [7,8]. The fibrotic response in IPF and PCPF is orchestrated in part by the immune system. The longer the insult acts on lung tissue, the lower the probability of complete resolution of lung damage. Among ILDs, sarcoidosis is a multisystemic inflammatory disorder that mainly affects the lungs, and its spontaneous resolution occurs in the early stages of disease, characterised by exaggerated immune cell activity. 

Immune responses are regulated by immune checkpoints (ICs), which are crucial for maintaining self-tolerance. Programmed cell death protein-1 (PD-1), also known as CD279, and an emerging immune checkpoint T cell immunoglobulin and ITIM domain (TIGIT) are both expressed on T cells. PD-1 and TIGIT are two T-cell exhaustion markers involved in different fibrotic mechanisms. Under different conditions, PD-1 can regulate cell activation, phagocytosis, migration, invasion of immune and non-immune cells (such as fibroblasts and epithelial cells), and epithelial-to-mesenchymal transition [9]. TIGIT can directly induce T-cell suppression by blocking their activation, proliferation, and acquisition of effector functions [10]. 

The aim of the present study was to analyse the similarities and differences in CD4^+^, CD8^+^, and NK cells in the peripheral blood of patients with fibrotic disease (IPF and PCPF) compared with sarcoidosis and healthy controls (HCs). A second aim was to evaluate expression and co-expression of PD-1 and TIGIT on CD4, CD8, and natural killer (NK) cells from our patient cohorts. 

## 2. Material and Methods

### 2.1. Study Population

We enrolled one hundred and fifteen patients with IPF, PCPF, and sarcoidosis monitored at the Rare Lung Disease Centre of Siena University Hospital. PCPF patients with comorbidities, including diabetes, heart failure, and hypertension, IPF patients with concomitant malignancies, and sarcoidosis patients with extrathoracic disease involvement and concomitant diseases were excluded. Patients who did not receive a diagnosis confirmation according to the international guidelines or PCPF patients without radiological confirmation of pulmonary fibrosis were excluded. 

The IPF diagnosis was confirmed by a multidisciplinary group according to international American Thoracic Society/European Respiratory Society (ATS/ERS) guidelines. Forty-eight IPF patients were enrolled before pharmacological treatment. Fifty-five PCPF patients were enrolled 6–9 months after discharge from hospital subsequent to admission with COVID-19 (hospitalised between March 2020 and May 2022) and returned to the regional program for disease monitoring. All PCPF patients underwent medical examination, including chest HRCT, blood tests, and lung function tests (LFTs). Twelve sarcoidosis patients were diagnosed according to international criteria based on clinical signs, chest radiography findings, and non-caseating granulomas in lymph nodes and/or endobronchial biopsy specimens. All patients with sarcoidosis enrolled in the study were in radiological stage II, characterised by mediastinal lymph node enlargement associated with micronodular lung parenchymal involvement. Ten healthy HCs without previous infectious or malignant diseases were enrolled as the HC group. Blood samples for immunological assay were drawn upon inclusion in the study. 

All patients gave their written informed consent for participation in the study. The study was approved by the regional ethical review board of Siena, Italy (C.E.A.V.S.E. Markerlung 17431), and complied with the declaration of Helsinki.

### 2.2. Peripheral Blood Mononuclear Cells

PBMC collection and management of cells was performed at the laboratory of the Respiratory Disease Unit, Siena University Hospital (Italy). The blood samples were drawn into a tube containing EDTA anticoagulant (BD Vacutainer^®^ EDTA tubes, BD Biosciences, CA, USA) and processed within 8 h. Subsequently, PBMCs were separated with gradient centrifugation (Ficoll Histopaque^®^-1077, Sigma-Aldrich, St. Louis, MO, USA) for 30 min at 1050 g without deceleration, then washed twice, resuspended in 80% RPMI 1640, 10% FBS, and 10% dimethyl sulfoxide (DMSO, Sigma-Aldrich, St. Louis, MO, USA) at 2 × 10^6^ cells per vial, and stored in liquid nitrogen until analysis.

### 2.3. Gating Strategy

Multicolour flow cytometric analysis was performed using mAb (Table 1).

**Figure 1:**
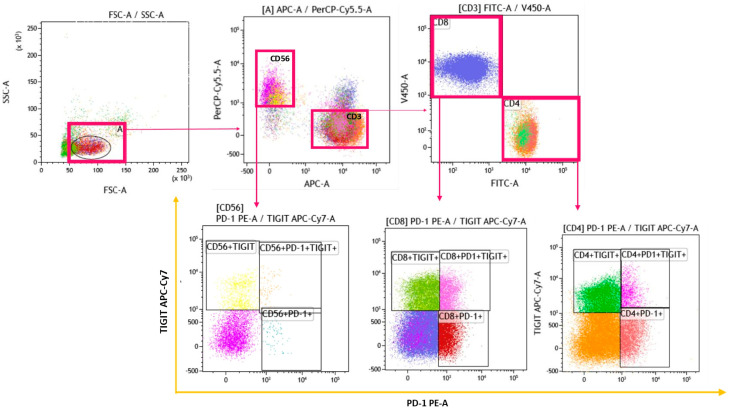
Lymphocytes were discriminated on the basis of forward (FSC) versus side (SSC) scatters. Then, a dot plot was performed to distinguished CD3- from CD56-expressing cells, and a second dot plot was performed to identify CD4 from CD8 cells. Using PD-1 and TIGIT markers, three dot plots were assessed on CD4-, CD8-, and CD56-positive cells to discriminate: CD4^+^PD-1^+^, CD4^+^TIGIT^+^, CD4^+^PD-1^+^ TIGIT^+^; CD8^+^PD-1^+^, CD8^+^TIGIT^+^, CD8^+^PD-1^+^TIGIT^+^; CD56^+^PD-1^+^, CD56^+^TIGIT^+^, CD56^+^PD-1^+^TIGIT^+^.

The gating strategy was performed with Kaluza Software 2.1 (Beckman Coulter, Brea, CA, USA). An assessment was conducted for T cell subsets and NK cells (Figure 1).

**Figure 2:**
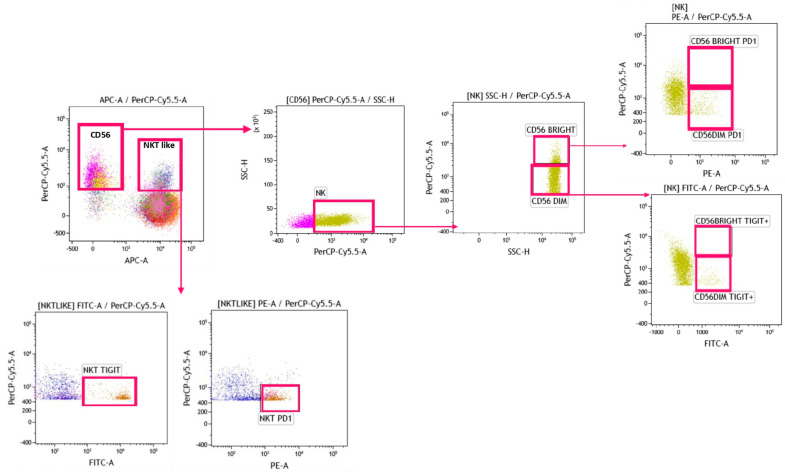
Two dot plots were performed to identify CD56^bright^ and CD56^dim^ expressing PD-1 or TIGIT. Further dot plots were performed to discriminate NKT-like cells expressing PD-1 and TIGIT.

Figure 2 reports the gating strategy performed with Kaluza Software 2.1 (Beckman Coulter, Brea, CA, USA) to identify CD56^dim^, CD56^bright^, and NKT-like cells. 

### 2.4. Lung Function Tests

The following lung function parameters were recorded according to standard ATS/ERS criteria using a Jaeger Body Plethysmograph with correction for temperature and barometric: forced expiratory volume in the first second (FEV1), forced vital capacity (FVC), carbon monoxide diffusing capacity (DLCO). All parameters were expressed as percentages of predicted values.

### 2.5. Statistical Analysis

All values were expressed as medians and interquartile ranges (IQRs) or means ± standard deviations when appropriate. A non-parametric one-way ANOVA (Kruskal–Wallis test) and the Dunn test were performed for multiple comparisons. The Spearman test was used to correlate immunological and clinical findings. A *p*-value less than 0.05 was considered statistically significant. Statistical analysis was performed using the GraphPad 10.1.2 and Jamovi 2.3.21 softwares. 

## 3. Results 

### 3.1. Study Population

Demographic and clinical data of patients and HCs are reported in Table 2.

We enrolled 48 patients with IPF (median age 73 (68–78) years; 73% males), 55 patients with PCPF (median age 75 (69–80) years; 53% males), 12 patients with sarcoidosis (median age 56 (54–58) years; 50% females), and 10 HCs (median age 69 (64–77) years; 50% males). IPF and PCPF patients were older than sarcoidosis patients (*p* = 0.003 and *p* = 0.001, respectively) and HCs (*p* < 0.001). A prevalence of smokers was found in the IPF cohort, whereas no-smokers were prevalent in PCPF patients (*p* < 0.001). No subject was on antifibrotic, steroid, or immunosuppressant treatment at the time of diagnosis.

### 3.2. Immunological Findings

Table 3 shows mean ± standard deviation and *p* values of cell subset percentages.

Matrix correlations, including all correlation data of immunological and lung function test parameters in HCs, sarcoidosis, IPF, and PCPF, are reported in Supplementary Materials (Figures S1–S4).

**Figure 3:**
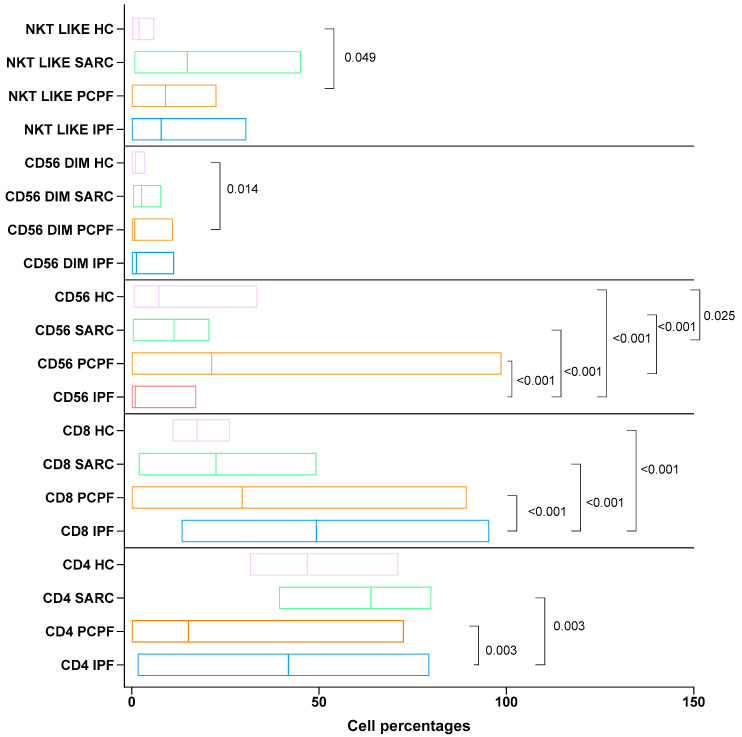
Comparison of CD4-, CD8-, and CD56-. CD56 ^DIM^ and NKT-like positive cell percentages in the three groups of diseases and healthy controls (HCs): IPF, idiopathic pulmonary fibrosis; PCPF, post-COVID-19 pulmonary fibrosis; SARC, sarcoidosis. Numerical values reported in the figure indicate the *p* values obtained via comparative analysis of groups: HCs, IPF, PCPF, and sarcoidosis.

Statistically significant differences in immunological findings between IPF, PCPF, sarcoidosis, and HC are reported in Figure 3. 

There was a direct correlation between CD4^+^ and CD8^+^ in PCPF patients (*p* < 0.001; r = 0.90), while in IPF and sarcoidosis patients, CD4^+^ and CD8^+^ were inversely correlated (*p* < 0.001 = −0.87 and *p* = 0.042; r = −0.6, respectively). 

**Figure 4:**
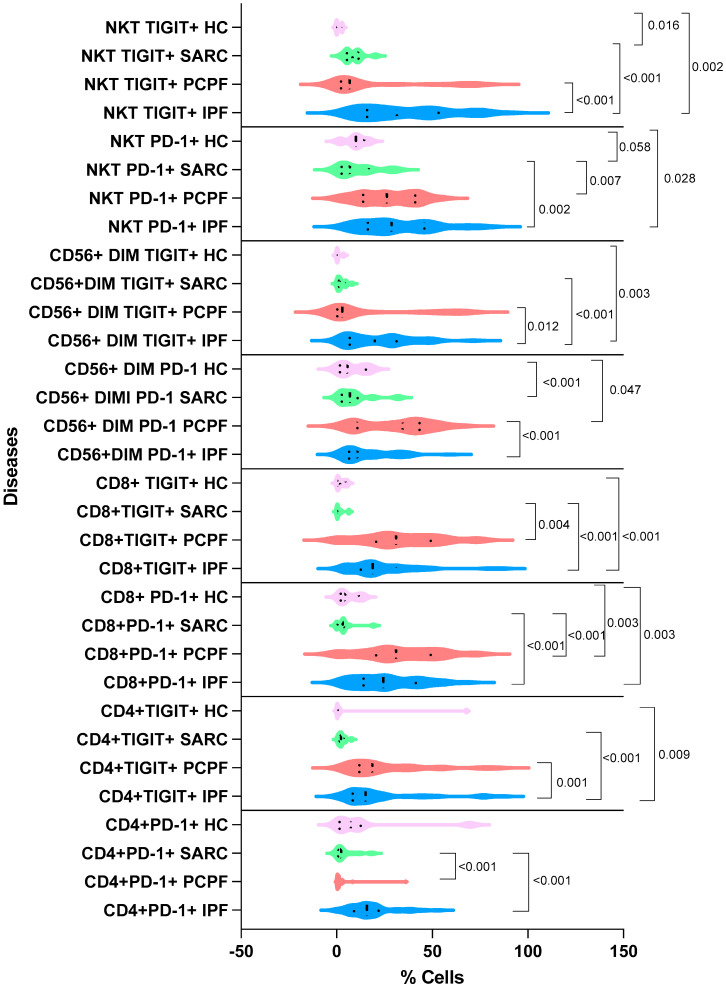
Comparison of CD4-, CD8-, and CD56-positive cell percentages expressing PD-1 and TIGIT in the three groups of diseases and healthy controls (HCs): IPF, idiopathic pulmonary fibrosis; SARC, sarcoidosis; PCPF, post-COVID-19 pulmonary fibrosis. Numerical values reported in the figure indicate the *p* values obtained via comparative analysis between HC, IPF, PCPF, and sarcoidosis groups.

Figure 4 shows the statistically significant comparative analysis of CD4-, CD8-, and CD56-expressed PD-1 and TIGIT in the four groups, IPF, PCPF, sarcoidosis, and HC.

A direct correlation was found in PCPF patients between CD4^+^ cells and CD8^+^, as well as PD-1^+^ and CD8^+^ TIGIT^+^ (r = 0.55, *p* < 0.001 and r = 0.55, *p* < 0.001, respectively).

Table 4 shows the mean ± standard deviation of percentages of CD4, CD8, and CD56 cells co-expressing PD-1 and TIGIT.

### 3.3. Lung Function Tests

IPF patients showed significantly lower FEV1(%) and FVC (%) than the PCPF (*p* = 0.016 and *p* < 0.001, respectively) and sarcoidosis cohorts (*p* = 0.041 and *p* = 0.001, respectively). Accordingly, we observed a significant reduction in DLCO (%) between the IPF cohort and PCPF and sarcoidosis patients (*p* < 0.001; *p* = 0.045, respectively).

## 4. Discussion

Here, we compared T and NK cell percentages in peripheral blood from IPF and PCPF patients with those of sarcoidosis and HC groups. CD4 and NK cells were more depleted in the IPF than in the other groups, even in normal ranges, in contrast with CD8 cells. Correlation analysis demonstrated an indirect correlation between CD4 and CD8 cells in IPF and sarcoidosis patients. Conversely, PCPF patients showed a direct correlation between CD4 and CD8 cells, highlighting the restoration of an immune response. 

Although data in the literature highlighted the similarities between post-COVID-19 syndrome and lung fibrosis, studies have reported the possible involvement of inflammatory cytokines, the renin–angiotensin system, the potential role of galectin-3, epithelial injuries in fibrosis, alveolar type 2 involvement, neutrophil extracellular traps, and other specific aspects (relationship with clinical and mechanical factors, epithelial transition mesenchymal, TGF-β signalling pathway, macrophages). Our study, for the first time, highlighted the similarities and differences between two fibrotic diseases, IPF and PCPF, in accordance with lymphocyte subsets and their functional phenotype.

Galati et al. showed a lack of significant differences in CD4 cell percentages between patients with IPF and healthy HCs [11], as confirmed by our results. Nevertheless, peripheral lymphopenia in IPF patients (with respect to those with other interstitial lung diseases (ILDs)) was confirmed to be a prognostic marker of disease progression [12]. This is the first time that researchers have compared two fibrotic diseases, IPF and PCPF, and demonstrated restoration of CD4 cells 6–9 months after SARS-CoV-2 infection. This finding suggests that lung fibrosis evolves differently after COVID-19 than in IPF, where it is chronic, progressive, and idiopathic.

The functional phenotype of T and NK cells in our patient cohort was demonstrated via expression of exhaustion T cell markers, PD-1, and TIGIT. Expression of PD-1 and TIGIT was abundant on T and NK cell subsets of the two lung fibrotic groups, IPF and PCPF. Likewise, co-expression of PD-1 and TIGIT on the surface of CD4 and CD8 cells increased in these diseases. IPF patients showed the highest co-expression of PD-1 and TIGIT on NK cells. Moreover, lung parameters showed more impaired respiratory function in IPF patients than in the PCPF and sarcoidosis groups. TIGIT expression was evaluated in our cohorts because it is a member of the second wave of IC receptors, which work in synergy with PD-1 [10]. Tumour-infiltrating T lymphocytes and NK cells express TIGIT in lung tissues. Increased expression of TIGIT on CD4 and CD8 cells observed in our IPF cohort may suggest recruitment of T cells from peripheral blood (this explains the peripheral lymphopenia of IPF patients) to the lung interstitium, as described in the histopathological features of IPF. 

Moreover, CD4 cells expressing PD-1 were higher in our fibrotic cohort (IPF and PCPF patients) than in the sarcoidosis group, suggesting involvement of PD-1 in fibrotic disorders but not granulomatous diseases. This is the first report of this finding, and it is in line with human models of lung fibrosis, where overexpression of CD4^+^PD1^+^ cells has been observed, suggesting dysregulation of immune checkpoint expression which influences the pathogenesis of IPF [13]. TIGIT is reported to be highly expressed on dysfunctional or exhausted T cells in chronic diseases such as chronic viral infection and cancer [14]. We observed lower percentages of CD4^+^- and CD8^+^-TIGIT^+^ in sarcoidosis patients than in our fibrotic groups, IPF and PCPF, suggesting that the latter disorders may express a higher degree of exhaustion of T cells. In exhausted CD4, CD8, and NK cells, several immune checkpoints were co-expressed with PD-1 and provided a synergistic inhibitory effect. In addition, TIGIT indicated more severe exhaustion. In line with a more severely exhausted phenotype, our data show a more impaired immune system in IPF than in sarcoidosis patients, as demonstrated by the highest CD4-, CD8-, and CD56-PD-1^+^ TIGIT^+^ cell percentages. 

Concerning CD8 cells, little data are available on their role in fibrotic diseases. Deng et al. suggested that they promote development of fibrosis in IPF through infiltration followed by differentiation into fibrotic tissues producing interleukins, such as Interferon-gamma (IFN-γ). Our results demonstrate higher CD8 cell percentages in IPF than in the other groups, inversely correlated with CD4 cell percentages, which are associated with severe lung injury demonstrated by low FEV1, FVC, and DLCO. Flow cytometry assessment of CD8 cell percentages could help physicians identify fibrotic patients in cases where ILD is suspected. Rha et al. found that a decrease in CD4 cells contributed to CD8 cell exhaustion in hospitalised COVID-19 patients [15]. Our study is the first to report a direct correlation between CD4 and the exhausted phenotype of CD8 cells expressing PD-1 and TIGIT in PCPF patients. This finding suggests that exhausted CD8+ cells may play a role in the active phase of COVID-19 and in long-term sequelae. In the stage II sarcoidosis patients (without pulmonary fibrosis) enrolled in the present study, lower CD8+PD-1+ cell percentages were found, in line with our previous original article [16]. 

Little is known about the biology of NK cells in the lungs, though NK cell percentages proved to be a potential marker of survival in IPF [17]. Our study is the first to compare peripheral CD56^dim^ and CD56^bright^ in two fibrotic lung diseases, IPF and PCPF. Lower percentages of CD56^bright^ were found in PCPF than in the HC group, unlike the mature phenotype (CD56^dim^), presumably due to the previous response to SARS-CoV-2 infection. 

The role of PD-1 in healthy NK cells was investigated by Esen et al. [18], who demonstrated reduced expression of IFN-γ and Tumour necrosis factor-α (TNF-α), as well as reduced degranulation. Moreover, despite their lower expression in PBMC, the subgroup of NK cells expressing PD-1 was CD56dim. In line with this study, our data showed abundant expression of PD-1 on CD56dim, mainly in PCPF patients with respect to HC. Quatrini et al. [19] suggested that PD-1 expression is not associated with NK cell exhaustion but rather with acute activation. This may explain why more robust PD-1 expression can be observed in NK cells that are stimulated, for example, by COVID-19. 

Studies on TIGIT expression in NK cells are limited. Faqrul Hasan et al. [20] suggested that expression of TIGIT is a marker of NK cell activation. However, chronic TIGIT engagement with its ligands in a tumour microenvironment leads to a functional decline in NK cells. In line with the literature, our results show that CD56dim TIGIT+ cells were more expressed in IPF patients, supporting the concept of immune system dysfunction [20]. In addition, NKT that expressed TIGIT were more numerous in IPF patients than in the other groups, which suggests a recruiter role of immune cells expressing TIGIT at the site of damage. Even though our study highlighted the similarities and differences between PCPF, IPF, and sarcoidosis compared with HCs, our study did not include a larger multicenter cohort, and PCPF needs to be investigated in a longer follow-up. These findings are worth being analysed in other biological fluids.

## 5. Conclusions

Our study shines a light on the immune responses involved in the development of pulmonary fibrosis, both idiopathic and secondary to SARS-CoV-2 infection. We only observed a significant imbalance in CD4, CD8, and NK percentages in peripheral blood from IPF and PCPF patients, but also in their functional phenotypes, evaluated through immune checkpoint expression. Our study confirmed immunological similarities between IPF and PCPF. Further study of these immunological pathways with a longer follow-up for PCPF patients would be worthwhile.

## Figures and Tables

**Table 1:** The features of monoclonal antibodies used for multicolour flow cytometric analysis, including clone, fluorochrome, and company.

Cluster of Differentiation (CD-)	Clone	Fluorochrome	Company
CD3	OKT3	APC	BioLegend (San Diego, CA, USA)
CD4	SK3	FITC	Becton Dickinson (Franklin Lakes, NJ, USA)
CD8	SK1	BV421	BioLegend
PD-1	PE	PD1.3.1.3	Miltenyi Biotec (Bergisch Gladbach, Germay)
TIGIT	APC-Cy7	A15153G	BioLegend
CD56	5.1H11	PerCP/Cy5.5	BioLegend

**Table 2:** Clinical and demographical data of enrolled patients. Data are reported as medians and ± standard deviations.

Clinical and Demographic Parameters	IPF (*n* = 48)	PCPF (*n* = 55)	Sarcoidosis (*n* = 12)	HC (*n* = 10)
Sex (F/M)	13/35	23/29	6/6	5/5
Age (median)	73 ± 8.11	75 ± 8.24	56 ± 6.76	69 ± 15.9
Smoking status (never/former)	41/7	52/3	5/7	10/0
Lung function test parameters (median and ±standard deviation):				
FEV1%	76.5 ± 20.42	94 ± 16.07	104 ± 10.09	
FVC%	73 ± 19.61	107 ± 10.98	110 ± 13.87	
DLCO%	45.5 ± 33.26	60 ± 12.35	76 ± 18.52	
Radiological findings	UIP (*n* = 48)	Fibrotic inter- or intralobular thickening (*n* = 55)/Air trapping (*n* = 43)/Groundglass (*n* = 40)	Scadding stage II (*n* = 12)	

**Table 3:** T and NK cell subset percentages with and without PD-1 and TIGIT expression divided according to disease group: IPF, idiopathic pulmonary fibrosis; PCPF, post-COVID-19 pulmonary fibrosis; sarcoidosis; and HCs, healthy controls. All data were reported as means ± standard deviations. Abbreviations: CD-, cluster of differentiation; NK, natural killer; NKT-like cells, natural killer like T cells; PD-1, programmed death-1; TIGIT, T cell immunoglobulin and ITIM domain.

Cell Percentages	IPF (*n* = 48)	PCPF (*n* = 55)	Sarcoidosis (*n* = 12)	HCs (*n* = 10)	*p* Value
CD3	49.0 ± 26.0	51.1 ± 22.3	41.3 ± 22.8	55.8 ± 7.58	0.561
CD4	41.8 ± 17.8	55.2 ± 18.6	63.8 ± 14.4	46.9 ± 12.2	<0.001
CD8	49.3 ± 18.5	28.4 ± 17.5	22.5 ± 12.8	17.4 ± 5.14	<0.001
CD4^+^PD-1^+^	17.9 ± 11.7	24.7 ± 19.9	4.37 ± 5.52	14.1 ± 23.0	<0.001
CD4^+^TIGIT^+^	22.3 ± 21.3	15.6 ± 23.2	2.73 ± 2.02	9.09 ± 23.7	<0.001
CD8^+^PD-1^+^	26.5 ± 16.9	33.5 ± 19.6	4.04 ± 5.16	6.25 ± 5.05	<0.001
CD8^+^TIGIT^+^	23.4 ± 17.5	30.3 ± 29.2	1.61 ± 2.30	2.40 ± 2.17	<0.001
CD56	0.922 ± 2.70	1.27 ± 1.38	11.3 ± 5.88	7.23 ± 10.9	<0.001
CD56^+^PD-1^+^	14.1 ± 20.9	12.6 ± 20.4	0.394 ± 1.11	7.32 ± 10.0	<0.001
CD56^+^TIGIT^+^	28.6 ± 24.3	22.9 ± 27.7	5.82 ± 6.13	7.73 ± 9.25	0.001
NK	45.8 ± 23.9	37.3 ± 19.0	46.1 ± 19.2	21.6 ± 22.3	0.044
CD56^bright^	3.33 ± 8.48	2.25 ± 3.49	4.70 ± 6.24	8.42 ± 3.35	0.029
CD56^dim^	95.7 ± 9.30	96.7 ± 14.1	92.2 ± 5.34	81.7 ± 6.53	0.005
CD56^bright^PD-1^+^	0.965 ± 6.65	1.36 ± 13.7	1.34 ± 0.879	0.360 ± 1.51	0.328
CD56^bright^TIGIT^+^	0.665 ± 5.92	0.480 ± 2.79	0.395 ± 0.561	0.0600 ± 1.58	0.076
CD56^dim^PD-1^+^	10.9 ± 13.2	34.4 ± 18.1	6.93 ± 8.88	5.73 ± 7.18	<0.001
CD56^dim^TIGIT^+^	19.9 ± 16.8	2.92 ± 25.7	1.94 ± 2.36	0.240 ± 1.50	<0.001
NKT-like cells	6.33 ± 6.94	7.97 ± 6.33	10.00 ± 14.9	0.180 ± 2.65	0.030
NKT PD-1^+^	28.6 ± 19.4	26.2 ± 14.9	6.93 ± 10.3	10.0 ± 5.43	<0.001
NKT TIGIT^+^	31.5 ± 23.7	6.83 ± 26.5	8.22 ± 4.81	0.00 ± 1.50	<0.001

**Table 4:** Co-expression of PD-1 and TIGIT on the surface of CD4, CD8, and CD56 cells. All data were expressed as means ± standard deviations. Lower CD4^+^PD-1^+^TIGIT^+^, CD8^+^PD-1^+^TIGIT^+^, and CD56^+^PD-1^+^TIGIT^+^ cell percentages were in HCs compared to IPF, PCPF, and sarcoidosis (*p* < 0.05). Further statistically significant differences in CD4^+^PD-1^+^TIGIT^+^ cell percentages were found between (a) IPF and sarcoidosis (*p* = 0.013). CD8^+^PD-1^+^TIGIT^+^ was higher in (b) IPF than in sarcoidosis (*p* < 0.001), as well as in (c) PCPF compared to IPF (mettere p) and sarcoidosis (*p* < 0.001). Higher CD56^+^PD-1^+^TIGIT^+^ cell percentages were in (d) IPF than in the PCPF and sarcoidosis groups (*p* = 0.038 and *p* = 0.005, respectively).

Double Positive Cell Percentages	IPF (*n* = 48)	PCPF (*n* = 55)	SARCOIDOSIS (*n* = 12)	HCs (*n* = 10)
CD4^+^PD-1^+^TIGIT^+^	6.21 ± 5.97 ^a^	8.67 ± 14.6	4.01 ± 9.83	0.0187 ± 0.0236
CD8^+^PD-1^+^TIGIT^+^	7.12 ± 8.12 ^b^	17.9 ± 20.3 ^c^	0.0709 ± 0.235	0.374 ± 0.716
CD56^+^PD-1^+^TIGIT^+^	15.7 ± 23.2 ^d^	10.5 ± 21.9	3.39 ± 6.64	1.61 ± 2.80

## Data Availability

The data presented in this study are available on request from the corresponding author.

## Supplementary Materials

The following supporting information can be downloaded at https://www.mdpi.com/article/10.3390/biomedicines12030630/s1. Figure S1. Matrix correlation of immunological and lung function test parameters in PCPF patients; Figure S2. Matrix correlation of immunological and lung function test parameters in IPF patients; Figure S3. Matrix correlation of immunological and lung function test parameters in sarcoidosis patients; Figure S4. Matrix correlation of immunological and lung function test parameters in healthy controls.

## Abbreviations

IPF, idiopathic pulmonary fibrosis; PCPF, post-COVID is pulmonary fibrosis; IC, immune checkpoint; HC, healthy control; TGF-β, Transforming growth factor beta; ECM, extracellular matrix; PD-1; Programmed cell death protein-1; TIGIT, T cell immunoglobulin and ITIM domain; NK, natural Killer; HRCT, highresolution computed tomography; IQR, interquartile range; p value, probability value; CD-, cluster of differentiation; ILD, interstitial lung disease; IFN-γ, Interferon-gamma; TNF-α, Tumor necrosis factor-α.
