# Supplementary material for: Immunological Similarities and Differences between Post-COVID-19 Lung Sequelae and Idiopathic Pulmonary Fibrosis
Source: Biomedicines. 2024 Mar 12;12(3):630. doi: 10.3390/biomedicines12030630 (PMC10967705; doi:10.3390/biomedicines12030630)

Figure s1. Matrix correlation of immunological and lung function test parameters in PCPF patients. The statistically significant correlations were reported in the manuscript.

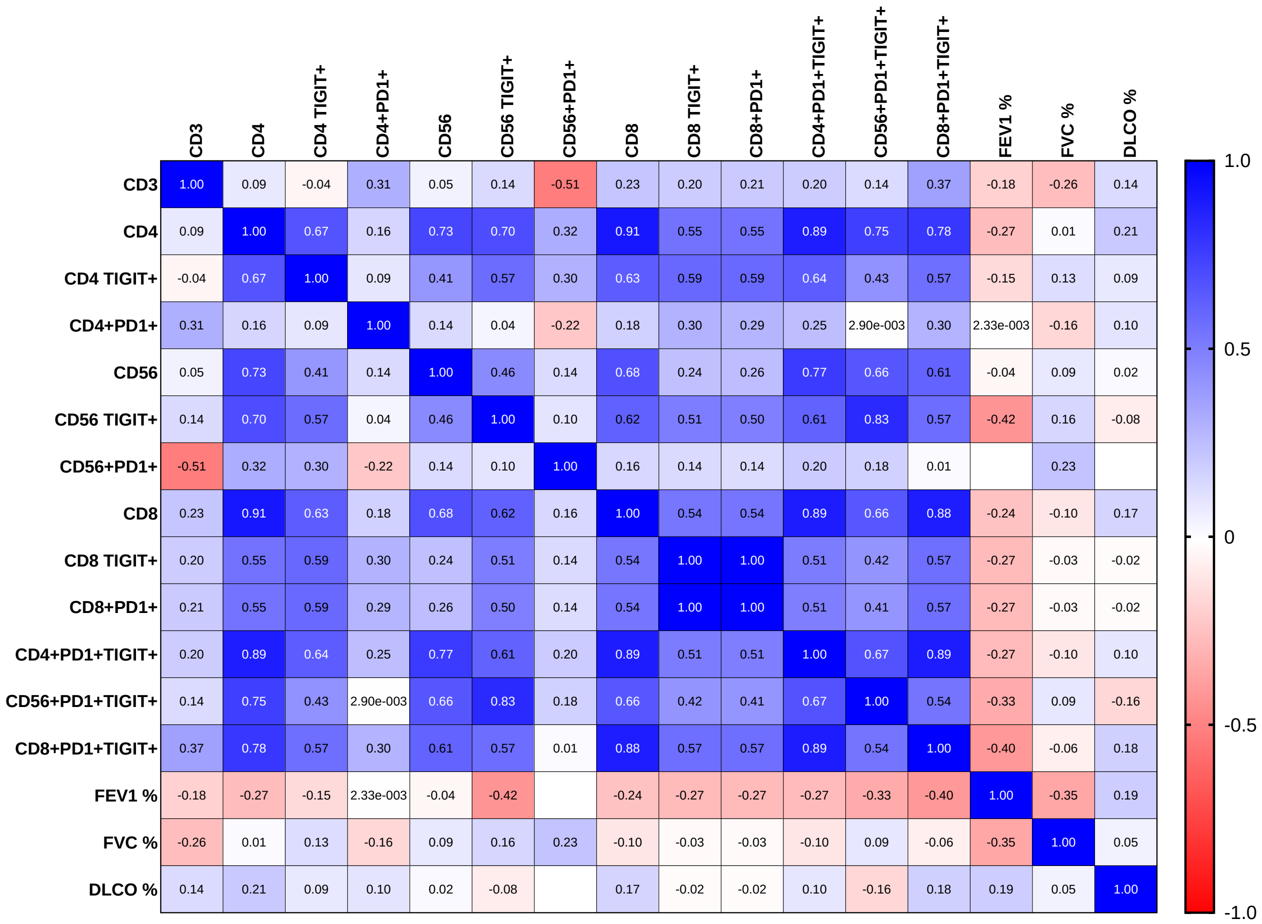


| | CD3 | CD4 | CD4 TIGIT+ | CD4+PD1+ | CD56 | CD56 TIGIT+ | CD56+PD1+ | CD8 | CD8 TIGIT+ | CD8+PD1+ | CD4+PD1+TIGIT+ | CD56+PD1+TIGIT+ | CD8+PD1+TIGIT+ | FEV1 % | FVC % | DLCO % |
|---|---|---|---|---|---|---|---|---|---|---|---|---|---|---|---|---|
| CD3 | 1.00 | 0.09 | -0.04 | 0.31 | 0.05 | 0.14 | -0.51 | 0.23 | 0.20 | 0.21 | 0.20 | 0.14 | 0.37 | -0.18 | -0.26 | 0.14 |
| CD4 | 0.09 | 1.00 | 0.67 | 0.16 | 0.73 | 0.70 | 0.32 | 0.91 | 0.55 | 0.55 | 0.89 | 0.75 | 0.78 | -0.27 | 0.01 | 0.21 |
| CD4 TIGIT+ | -0.04 | 0.67 | 1.00 | 0.09 | 0.41 | 0.57 | 0.30 | 0.63 | 0.59 | 0.59 | 0.64 | 0.43 | 0.57 | -0.15 | 0.13 | 0.09 |
| CD4+PD1+ | 0.31 | 0.16 | 0.09 | 1.00 | 0.14 | 0.04 | -0.22 | 0.18 | 0.30 | 0.29 | 0.25 | 2.90e-003 | 0.30 | 2.33e-003 | -0.16 | 0.10 |
| CD56 | 0.05 | 0.73 | 0.41 | 0.14 | 1.00 | 0.46 | 0.14 | 0.68 | 0.24 | 0.26 | 0.77 | 0.66 | 0.61 | -0.04 | 0.09 | 0.02 |
| CD56 TIGIT+ | 0.14 | 0.70 | 0.57 | 0.04 | 0.46 | 1.00 | 0.10 | 0.62 | 0.51 | 0.50 | 0.61 | 0.83 | 0.57 | -0.42 | 0.16 | -0.08 |
| CD56+PD1+ | -0.51 | 0.32 | 0.30 | -0.22 | 0.14 | 0.10 | 1.00 | 0.16 | 0.14 | 0.14 | 0.20 | 0.18 | 0.01 | | 0.23 | |
| CD8 | 0.23 | 0.91 | 0.63 | 0.18 | 0.68 | 0.62 | 0.16 | 1.00 | 0.54 | 0.54 | 0.89 | 0.66 | 0.88 | -0.24 | -0.10 | 0.17 |
| CD8 TIGIT+ | 0.20 | 0.55 | 0.59 | 0.30 | 0.24 | 0.51 | 0.14 | 0.54 | 1.00 | 1.00 | 0.51 | 0.42 | 0.57 | -0.27 | -0.03 | -0.02 |
| CD8+PD1+ | 0.21 | 0.55 | 0.59 | 0.29 | 0.26 | 0.50 | 0.14 | 0.54 | 1.00 | 1.00 | 0.51 | 0.41 | 0.57 | -0.27 | -0.03 | -0.02 |
| CD4+PD1+TIGIT+ | 0.20 | 0.89 | 0.64 | 0.25 | 0.77 | 0.61 | 0.20 | 0.89 | 0.51 | 0.51 | 1.00 | 0.67 | 0.89 | -0.27 | -0.10 | 0.10 |
| CD56+PD1+TIGIT+ | 0.14 | 0.75 | 0.43 | 2.90e-003 | 0.66 | 0.83 | 0.18 | 0.66 | 0.42 | 0.41 | 0.67 | 1.00 | 0.54 | -0.33 | 0.09 | -0.16 |
| CD8+PD1+TIGIT+ | 0.37 | 0.78 | 0.57 | 0.30 | 0.61 | 0.57 | 0.01 | 0.88 | 0.57 | 0.57 | 0.89 | 0.54 | 1.00 | -0.40 | -0.06 | 0.18 |
| FEV1 % | -0.18 | -0.27 | -0.15 | 2.33e-003 | -0.04 | -0.42 | | -0.24 | -0.27 | -0.27 | -0.27 | -0.33 | -0.40 | 1.00 | -0.35 | 0.19 |
| FVC % | -0.26 | 0.01 | 0.13 | -0.16 | 0.09 | 0.16 | 0.23 | -0.10 | -0.03 | -0.03 | -0.10 | 0.09 | -0.06 | -0.35 | 1.00 | 0.05 |
| DLCO % | 0.14 | 0.21 | 0.09 | 0.10 | 0.02 | -0.08 | | 0.17 | -0.02 | -0.02 | 0.10 | -0.16 | 0.18 | 0.19 | 0.05 | 1.00 |

Figure s2. Matrix correlation of immunological and lung function test parameters in IPF patients. The statistically significant correlations were reported in the manuscript.

| | CD3 | CD4 | CD4 TIGIT+ | CD4+PD1+ | CD56 | CD56 TIGIT+ | CD56+PD1+ | CD8 | CD8 TIGIT+ | CD8+PD1+ | CD4+PD1+TIGIT+ | CD56+PD1+TIGIT+ | CD8+PD1+TIGIT+ | FEV1 % | FVC % | DLCO % |
|---|---|---|---|---|---|---|---|---|---|---|---|---|---|---|---|---|
| **CD3** | 1.00 | 0.01 | -0.37 | 0.10 | -0.07 | -0.24 | -0.34 | 0.14 | -0.25 | 0.01 | -0.16 | -0.40 | -0.12 | -0.25 | -0.20 | -0.06 |
| **CD4** | 0.01 | 1.00 | 0.10 | -0.20 | 0.18 | 4.45e-003 | -0.12 | -0.87 | -0.01 | 0.25 | 0.20 | -0.04 | 0.21 | 0.02 | -0.09 | -0.01 |
| **CD4 TIGIT+** | -0.37 | 0.10 | 1.00 | 0.10 | -0.32 | 0.51 | 0.22 | -0.21 | 0.75 | 0.20 | 0.52 | 0.46 | 0.50 | -0.01 | -0.08 | -0.11 |
| **CD4+PD1+** | 0.10 | -0.20 | 0.10 | 1.00 | 0.08 | 0.05 | 0.05 | 0.12 | 0.05 | 0.46 | 0.54 | 0.08 | 0.26 | -0.03 | -0.07 | 0.26 |
| **CD56** | -0.07 | 0.18 | -0.32 | 0.08 | 1.00 | -0.03 | -0.31 | -0.11 | -0.22 | 0.15 | -0.01 | -0.32 | 0.06 | 0.25 | 0.25 | 0.14 |
| **CD56 TIGIT+** | -0.24 | 4.45e-003 | 0.51 | 0.05 | -0.03 | 1.00 | 0.55 | 0.10 | 0.43 | 0.02 | 0.27 | 0.80 | 0.24 | -0.04 | -0.02 | 0.03 |
| **CD56+PD1+** | -0.34 | -0.12 | 0.22 | 0.05 | -0.31 | 0.55 | 1.00 | 0.04 | 0.34 | 0.13 | 0.35 | 0.69 | 0.24 | 0.01 | 0.04 | 0.19 |
| **CD8** | 0.14 | -0.87 | -0.21 | 0.12 | -0.11 | 0.10 | 0.04 | 1.00 | -0.11 | -0.23 | -0.27 | 0.02 | -0.23 | -0.10 | 0.04 | 0.03 |
| **CD8 TIGIT+** | -0.25 | -0.01 | 0.75 | 0.05 | -0.22 | 0.43 | 0.34 | -0.11 | 1.00 | 0.20 | 0.56 | 0.34 | 0.67 | -0.09 | -0.16 | -0.15 |
| **CD8+PD1+** | 0.01 | 0.25 | 0.20 | 0.46 | 0.15 | 0.02 | 0.13 | -0.23 | 0.20 | 1.00 | 0.59 | -0.04 | 0.72 | 0.03 | -0.02 | 0.13 |
| **CD4+PD1+TIGIT+** | -0.16 | 0.20 | 0.52 | 0.54 | -0.01 | 0.27 | 0.35 | -0.27 | 0.56 | 0.59 | 1.00 | 0.33 | 0.72 | -0.20 | -0.21 | -0.01 |
| **CD56+PD1+TIGIT+** | -0.40 | -0.04 | 0.46 | 0.08 | -0.32 | 0.80 | 0.69 | 0.02 | 0.34 | -0.04 | 0.33 | 1.00 | 0.11 | 0.04 | 0.10 | 0.02 |
| **CD8+PD1+TIGIT+** | -0.12 | 0.21 | 0.50 | 0.26 | 0.06 | 0.24 | 0.24 | -0.23 | 0.67 | 0.72 | 0.72 | 0.11 | 1.00 | -0.06 | -0.07 | 0.06 |
| **FEV1 %** | -0.25 | 0.02 | -0.01 | -0.03 | 0.25 | -0.04 | 0.01 | -0.10 | -0.09 | 0.03 | -0.20 | 0.04 | -0.06 | 1.00 | 0.91 | 0.49 |
| **FVC %** | -0.20 | -0.09 | -0.08 | -0.07 | 0.25 | -0.02 | 0.04 | 0.04 | -0.16 | -0.02 | -0.21 | 0.10 | -0.07 | 0.91 | 1.00 | 0.36 |
| **DLCO %** | -0.06 | -0.01 | -0.11 | 0.26 | 0.14 | 0.03 | 0.19 | 0.03 | -0.15 | 0.13 | -0.01 | 0.02 | 0.06 | 0.49 | 0.36 | 1.00 |

1.0 0.5 0 -0.5 -1.0

Figure s3. Matrix correlation of immunological and lung function test parameters in sarcoidosis patients. The statistically significant correlations were reported in the manuscript.

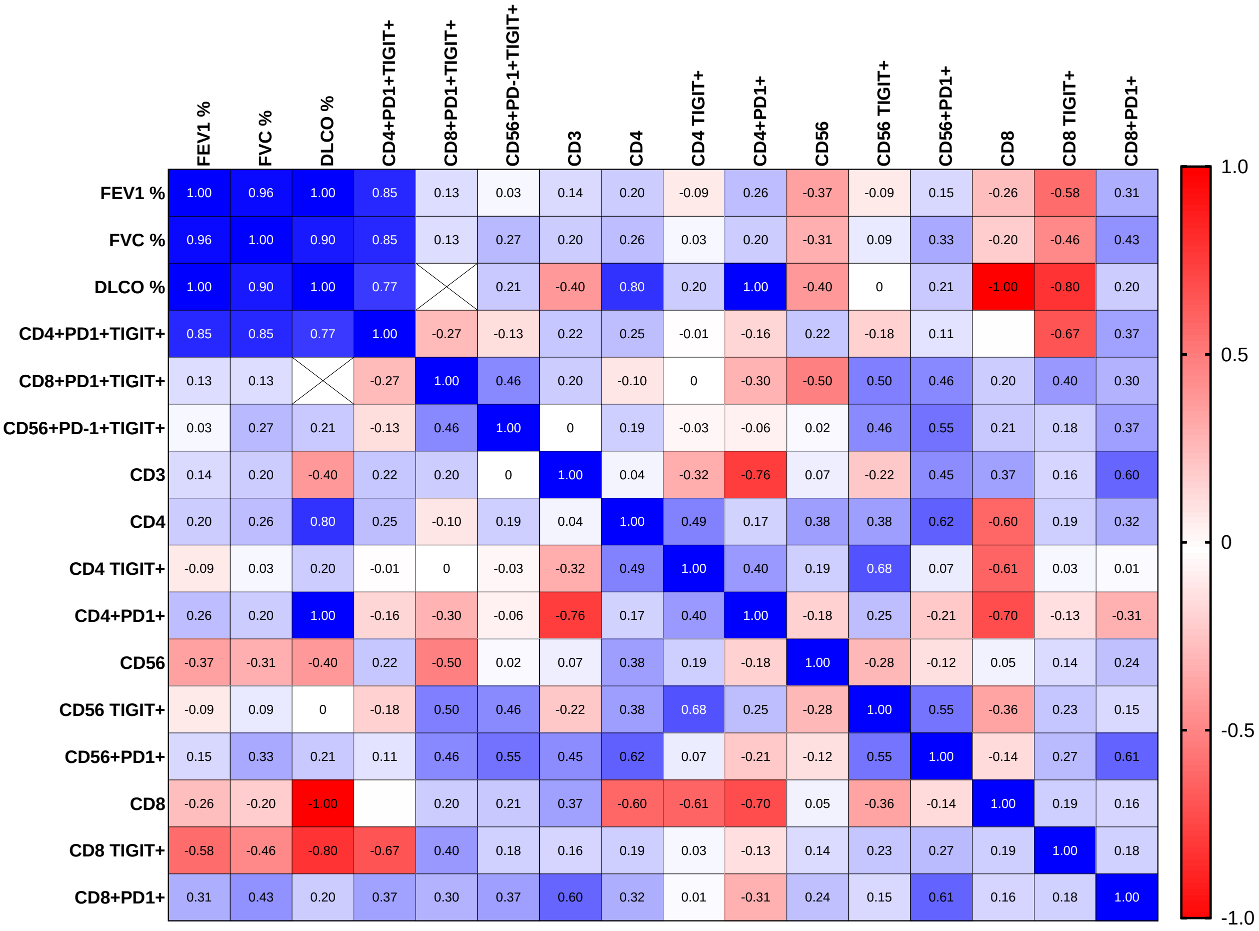

Figure s4. Matrix correlation of immunological and lung function test parameters in healthy controls. The statistically significant correlations were reported in the manuscript.

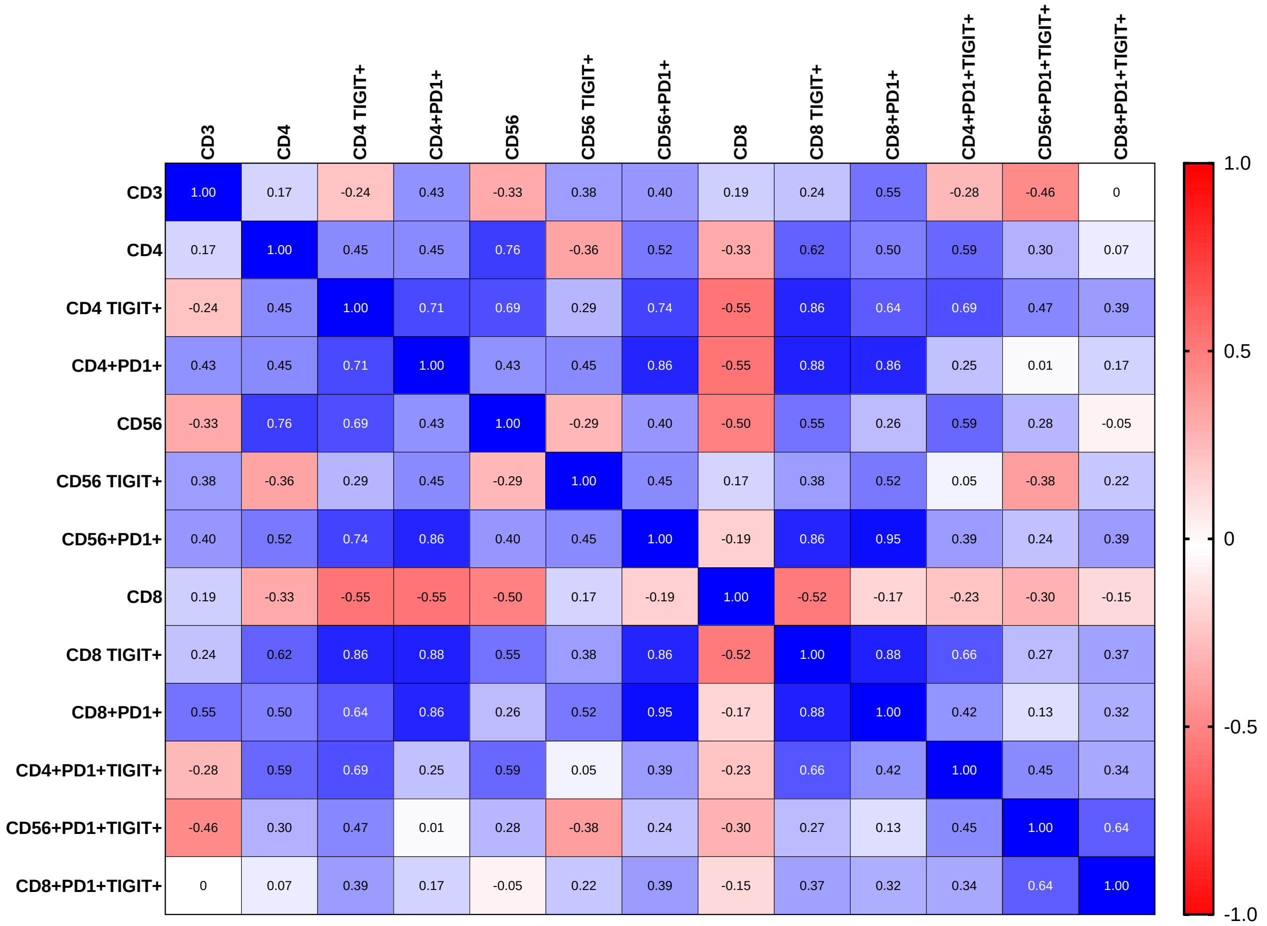

Supplement: Supplementary file 1 [file biomedicines-12-00630-s001.zip › biomedicines-2864437-supplementary.pdf]
